# Adherence to NICE guidance on lifestyle advice for people with schizophrenia: a survey

**DOI:** 10.1192/pb.bp.116.054304

**Published:** 2017-06

**Authors:** Lizzie Swaby, Daniel Hind, Rebecca Gossage-Worrall, David Shiers, Jonathan Mitchell, Richard I. G. Holt

**Affiliations:** 1 University of Sheffield; 2 Greater Manchester West Mental Health NHS Foundation Trust; 3 Sheffield Health and Social Care NHS Foundation Trust; 4 University of Southampton

## Abstract

**Aims and method** The STEPWISE trial (STructured lifestyle Education for People WIth SchizophrEnia, schizoaffective disorder and first episode psychosis) is currently evaluating a lifestyle education programme in addition to usual care. However, it is difficult to define what constitutes ‘usual care’. We aimed to define ‘usual care’ for lifestyle management in people with schizophrenia, schizoaffective disorder and first-episode psychosis in STEPWISE study sites. Ten National Health Service (NHS) mental health trusts participated in a bespoke survey based on the National Institute for Health and Care Excellence (NICE) guidance.

**Results** Eight trusts reported offering lifestyle education programmes and nine offered smoking cessation support. Reported recording of biomedical measures varied.

**Clinical implications** Although recommended by NICE, lifestyle education programmes are not consistently offered across UK NHS mental health trusts. This highlights missed opportunities to improve the physical health of people with psychotic illness. Our survey benchmarks ‘usual care’ for the STEPWISE study, against which changes can be measured. Furthermore, future studies will be able to identify whether any progress in clinical practice has been made towards achieving the NICE recommendations.

The prevalence of overweight (body mass index (BMI) >25 kg/m^2^) and obesity (BMI>30 kg/m^2^) in adults with schizophrenia is approximately twice that in the general population and while it has increased in both groups in the past 30 years, the rate of increase is greater in people with schizophrenia.^1^ Weight gain often occurs after initiation of antipsychotic treatment: over 50% of individuals gain more than 7% of their initial body weight within the first 12 months of treatment, with up to 86% gaining weight with some types of antipsychotic.^2^ Compared with the general population, people with schizophrenia consume more fat and refined carbohydrates and fewer vegetables,^3–5^ are less physically active and experience higher levels of deprivation,^4–7^ all of which are associated with obesity. The National Institute for Health and Care Excellence (NICE) recommends that people with psychosis or schizophrenia, particularly those taking antipsychotics, should be offered access to a combined healthy eating and physical activity programme to aid in the prevention of weight gain and its related comorbidities, leading to improved quality of life.^8^ They should be supported by clinicians to make choices about antipsychotic medication, informed by discussions of likely benefits, possible side-effects, such as weight gain and metabolic disturbance, and impact on other aspects of their physical health. For those who smoke, help should be offered to stop smoking.^8^

The STEPWISE study (STructured lifestyle Education for People WIth SchizophrEnia, schizoaffective disorder and first episode psychosis; ISRCTN19447796) was commissioned to evaluate the extent to which a structured lifestyle education programme can support weight loss, compared with usual care, when delivered to adults with schizophrenia, including those with schizoaffective disorder or first-episode psychosis, in a community mental health setting. In pragmatic trials, practitioners are often allowed ‘considerable leeway in deciding how to formulate and apply’ the treatment in a control arm defined as ‘usual care’.^9^ While the result may be reflective of the care received by patients outside the trial, there is often variation in care in an active control arm, which may be difficult to document.^10,11^ For this reason, study teams often try to capture the active content of the control arm of their trials.^12^ In this paper we describe a survey of ‘usual care’ for the management of weight and other lifestyle factors in people with schizophrenia, schizoaffective disorder and first-episode psychosis in the ten participating study sites.

## Method

### Research tool

The survey instrument consisted of 14 questions, which were a combination of closed and open design, with the aim of eliciting information about the implementation of the recent NICE guidance on psychosis and schizophrenia at National Health Service (NHS) mental health trusts in the UK, and consequently what is offered as ‘usual care’. The survey was developed for the research study, based on the physical health aspects of the NICE recommendations in clinical guideline 178.^8^ Questions followed the baseline assessment tool published alongside the NICE guidance. The instrument was piloted with one study site investigator (J.M.) on two occasions, and each led to substantial changes to the tool. For instance, the second iteration revised questions to ascertain proportions of patients to whom certain criteria apply, rather than closed questions such as ‘Are patients referred to a healthy eating programme?’ The final version of the tool also clarified which questions related to all patients with schizophrenia, schizoaffective disorder and first-episode psychosis, and which questions related to patients with first-episode psychosis only (the questionnaire is included in the Appendix). This is in line with the additional recommendations specified in the NICE guidance for patients with first-episode psychosis who have recently been prescribed antipsychotic medication; these additional questions were not applicable to the management of patients with established mental illnesses.

### Survey structure

Trusts were initially asked whether they offered a healthy eating and physical activity programme. Subsequent questions asked for more information about these programmes, including whether they were combined or separate for healthy eating and physical activity; how patients accessed them; and how often patients were referred to such services. Additional information was sought on the availability of smoking cessation services in this population and whether discussions took place between patients and clinicians prior to antipsychotic treatment initiation, including benefits of treatment, interactions with other substances and other possible side-effects.

In addition, NICE recommend that a number of physiological measures should be recorded both prior to the patient starting antipsychotic medication and annually thereafter. Respondents were asked to comment on how likely it was that each measure would be recorded at both of these time points in their trust and how often patients on antipsychotic medication have their weight recorded.

### Sample selection

The STEPWISE trial has ten centres in a variety of urban, suburban and rural locations across England: Sheffield Health and Social Care NHS Foundation Trust, Leeds and York Partnership NHS Foundation Trust, Bradford District Care NHS Foundation Trust, Greater Manchester West Mental Health NHS Foundation Trust, South London and Maudsley NHS Foundation Trust, Sussex Partnership NHS Foundation Trust, Southern Health NHS Foundation Trust, Devon Partnership NHS Trust, Somerset Partnership NHS Foundation Trust and Cornwall Partnership NHS Foundation Trust. A representative from each centre was invited to complete the survey.

### Respondents

The principal investigators at each of the ten centres for the STEPWISE trial were initially approached. Some of them completed the survey themselves, while others delegated to trust physical health leads or equivalent as they were better placed to answer the questions. Contact was made via email in the first instance, with an invitation to attend a teleconference with the STEPWISE research assistant (L.S.) to complete the survey. Those who did not provide a response to the invitation within 4 weeks were contacted again by reminder emails and/or by telephone. Six of the ten sites' surveys were completed through discussion via teleconference. The remaining four sites' surveys were completed independently by a trust representative and written responses were provided to the STEPWISE research assistant.

No sites required more than one reminder email/telephone call in order to arrange completion of the survey. As the survey information was requested from members of a research team, a favourable ethical opinion from an NHS Research Ethics Committee was not sought and consent was unnecessary. Responses provided organisational data only and did not include any personal data. Responses from all sites were received between 22 May and 28 October 2015.

### Analysis

Descriptive statistics in the form of counts were produced for quantitative variables. Supporting information provided by respondents and information yielded from qualitative questions was summarised in narrative form.

## Results

### Healthy eating and physical activity programmes

Eight respondents reported that their trust offered programmes on healthy eating and physical activity, which were mostly separate programmes. Supplementary information indicated that provision was *ad hoc* and interventions were rarely standardised. Most respondents reported routinely inviting patients to access services such as discounted local gym memberships, cooking groups and activity groups delivered by local authorities and third-sector organisations. Two trusts reported offering one-on-one advice sessions with healthy living advisors or health trainers, but the sessions were usually advice-giving and often more clinically focused rather than looking at the patient's physical well-being.

Those trusts that offered trust-led programmes reported that these were available in principle to all of their patients rather than specific groups based on diagnosis. However, interventions were often accessed only by certain groups of patients, usually through particular clinicians or clinics. One trust estimated that 70% of their eligible patients are referred to such services by mental health professionals, based on recent Commissioning for Quality and Innovation (CQUIN) results; other respondents were unaware of routine data from which they could quantify referrals. Six trusts reported offering lifestyle advice through open-ended group courses, three through courses delivered over a fixed period and four through drop-in sessions.

### Smoking cessation advice

Six respondents indicated that patients who smoke were offered help to stop some of the time, three reported help was offered all of the time, and one said this was not offered at all. Respondents who selected ‘some of the time’ were unable to quantify this, but felt that this occurred most of the time. Although it varied whether smoking cessation services were offered by the trusts or external services, most offered a combination of the two. Seven trusts reported offering trust-led smoking cessation services, while others had trained smoking cessation advisors but had no formal trust-offered service. Most trusts reported signposting outpatients to external services, some of which were managed by primary care, with advice leaflets available within the trust.

### Antipsychotic treatment initiation

Table 1 shows the reported levels of discussion about likely benefits of treatment, as well as potential weight gain, diabetes and metabolic side-effects and any other possible side-effects, across all respondents. Most trusts who reported that the recommended discussions took place ‘some of the time’ felt that this would be most of the time, but there was a lack of evidence to support this. One site reported that discussions would be dependent on the clinician, but that resources were available to clinicians to support the discussions. Another site suggested that such discussions may be part of an ongoing process rather than all happening in one session, depending on the patient's level of capacity.

**Table 1:** Discussions with patients when deciding on antipsychotic treatment (*n* = 10 NHS trusts)

	All of the time	Some of the time	Not at all
Topic			
Likely benefits	7	3	0
Weight gain	5	5	0
Diabetes and metabolic side-effects	3	7	0
Other possible side-effects	5	5	0

Other substances			
Alcohol	5	5	0
Tobacco	2	8	0
Other prescribed medications	3	7	0
Non-prescribed medications	0	10	0
Illicit drugs	3	7	0

Table 1 also shows how often respondents reported discussions taking place regarding the use of alcohol, tobacco, prescribed and non-prescribed medications and illicit drugs, at the time of antipsychotic treatment initiation.

Table 2 shows how likely trusts considered that physiological measures would be recorded prior to treatment initiation.

**Table 2:** Recording of physiological measures prior to antipsychotic treatment initiation (*n* = 10 NHS trusts)

	Very likely	Likely	Neither likely nor unlikely	Unlikely	Very unlikely
Weight	3	4	1	2	0

Weight plotted on a chart	1	3	1	3	2

Waist circumference	0	2	3	3	2

Pulse	3	4	1	1	1

Blood pressure	4	2	1	2	1

Fasting blood glucose	0	5	1	2	2

Random blood glucose	2	4	0	3	1

HbA1c	2	2	1	2	3

Blood lipid profile	2	4	0	2	2

Assessment of any movement disorders	2	4	2	0	2

Assessment of nutritional status, diet and level of physical activity	3	3	1	3	0

### Ongoing monitoring of weight and other physiological measures

It was clear from the responses that there were variations in recording patients' weight at the time points recommended by NICE (first at 6 weeks post-treatment initiation, then at 12 weeks, at 1 year and annually thereafter), both between trusts and within trust services. Some confusion exists regarding responsibility for annual patient reviews in the community and whether these should be completed by the general practitioner (GP) or the trust. Half of those surveyed reported that it was neither likely nor unlikely that patients would have their weight recorded weekly for the first 6 weeks, with three other trusts reporting that this was very unlikely. There was an even spread across all response categories as to whether weight was recorded at 12 weeks, but at 1 year four of those surveyed reported that patients were very likely to have their weight recorded. Six respondents reported that weight was likely or very likely to be recorded annually thereafter, although this was where the confusion arose regarding responsibility for these reviews.

Table 3 shows trusts' consideration as to how likely it was that physiological measures would be recorded at least annually in patients taking antipsychotic medication. One site felt they were unable to answer this question, so we present data reflecting responses from nine trusts.

**Table 3:** Recording of physiological measures at least annually for patients on antipsychotic medication (*n* = 9 NHS trusts)

	Very likely	Likely	Neither likely or unlikely	Unlikely	Very unlikely
Weight	3	4	0	2	0

Weight plotted on a chart	0	3	3	2	1

Waist circumference	0	2	2	3	2

Pulse	2	4	1	1	1

Blood pressure	3	4	1	1	0

Fasting blood glucose	1	2	5	1	0

HbA1c	2	2	4	1	0

Blood lipid profile	2	1	4	2	0

Assessment of any movement disorders	1	4	3	1	0

Assessment of nutritional status, diet and level of physical activity	3	1	3	2	0

There was no correlation between which of the recommended physiological measures were recorded by trusts prior to antipsychotic treatment initiation or annually thereafter, although generally those trusts who were likely to record particular measures prior to treatment initiation were also likely to record the same measures at least annually thereafter.

## Discussion

### Principal findings

It was clear from this survey that there was great variation between different NHS mental health trusts in the provision of healthy eating and physical activity interventions routinely offered to patients, as well as variation between clinicians within the same trust, with interventions often accessed only through particular clinicians or clinics. Commonly, trusts reported signposting or referring patients to programmes offered by external services, such as gym memberships and activity classes.

Most patients had access to a smoking cessation service should they require it, and referrals to such services were reported to occur most if not all of the time at nine out of the ten trusts surveyed, regardless of whether the service was offered within the trust or run externally.

When deciding on antipsychotic medication with newly diagnosed patients, there was also variability in the reported discussions that took place across trusts. All trusts reported that the likely benefits, weight gain, diabetes and metabolic and other possible side-effects were discussed with the patient at least some of the time; the possible interference of other substances with prescribed antipsychotic medication was also discussed.

Although at the early stages in the course of antipsychotic treatment it was reported unlikely that trusts would record a patient's weight, as recommended by NICE, by 1 year after treatment initiation a larger proportion of trusts reported weight recording, with an increase for annual reviews thereafter, despite the uncertainties regarding responsibility for undertaking annual reviews in the community.

### Study strengths and limitations

A strength of this survey was that its design was based on the NICE guidelines to which mental health trusts should be adhering. This meant that trusts' compliance with these recommendations could be assessed, allowing us to elicit information on what programmes (if any) trusts were offering in usual practice and how these compared with what is recommended by NICE. This also allowed us to try to define ‘usual care’ in relation to the STEPWISE study, using a standard approach across all trusts.

The survey was, in principle, a suitable method to elicit the same information from all respondents; however, it was clear from the responses that owing to the variability of services offered it was often difficult to provide a succinct account using this survey tool. The narrative information provided by the respondents proved more useful in gaining a fuller picture of their usual care than the descriptive statistics, which in some cases were a best guess, as clear data were not always available.

Furthermore, responses were based on one member of staff's knowledge of usual care in practice, and although this person was usually best placed to answer the questions, from the survey responses received knowledge may have been limited, especially as some interventions offered were particular to a specific clinic or clinician and usual care may vary within and between community mental health teams in any given NHS organisation. In addition, how representative ‘usual care’ is in comparison with NHS trusts not taking part in the STEPWISE study remains unknown.

The levels of detail regarding the content of available services also varied, perhaps indicating that the respondent had more involvement with some programmes than others. Therefore, it was considered likely that additional interventions may have been offered within trusts of which the survey respondent was unaware.

In relation to implementation, a weakness in the survey was highlighted when some responses were received through telephone discussion while others were completed by the respondent and returned to the researcher. No systematic differences between telephone and paper copy responses were identified, although more supporting information was often provided through telephone responses, as these were elicited through more of a conversational discussion. This difference in response methods may have caused questions to be perceived differently, although all telephone participants had a copy of the questionnaire available to them at the time of completion. Perceptions of appropriate levels of detail can change with different methods of completion, which may lead to variation in results. However, as such variation was evident between practices offered as ‘usual care’ in the ten trusts surveyed, the impact of these differences in completion method is considered likely to be small.

The variability in the information elicited has not allowed for a common picture across all sites, as although a type of programme is recommended by NICE, a particular standardised programme is not available across all trusts. However, the survey did provide sufficient baseline information to allow any changes in usual practice during the course of the STEPWISE study to be monitored at a trust level, rather than across all study sites as a whole. This will enable assessing at the end of the STEPWISE study the extent of potential contamination between the intervention and control arms of the trial, based on changes in practice reported in the survey.

### Context

Although there may be an increased awareness of the potential benefits of some treatments, this does not ensure that such treatments are implemented effectively. Evaluations and methods of improving the implementation of NICE guidelines often have limited attention.^13^ A systematic review undertaken by Berry & Haddock^13^ noted that the research around the implementation of NICE guidelines on schizophrenia is relatively limited, suggesting that these patients have poor access to psychological interventions such as cognitive-behavioural therapy (CBT). Some barriers to implementation of NICE guidelines were reported, such as insufficient support from trust management and the needs of organisations. The paper also highlights that whereas NICE considers randomised controlled trials (RCTs) to be the gold standard when developing an evidence base for its guidelines, RCTs have also been criticised for their poor ability to reflect the ‘real world’. The authors suggest that targeting these barriers is key to facilitating successful implementation of the guidelines.^13^

It is therefore important to identify which aspects of the guidance are not currently being followed, in order to target these areas for implementation and improve clinical care. Not only is it important to consider the implementation of guidance relating to monitoring of biomedical measures, but there is also likely to be a limited effect unless this is combined with sufficient intervention in behaviour or treatment. Similarly, the mere fact of guidance or a trial does not necessarily lead to substantive changes or better outcomes. Repeating the survey annually will allow identification of any substantive, systematic changes within the organisations, both since the introduction of the NICE guidelines and throughout the duration of the STEPWISE trial.

From a research perspective, the reported variation also has implications for our study when defining ‘usual care’. If all trusts adhered to all recommendations in the NICE guidelines, we could be sufficiently confident that contamination between trial arms would be minimal. However, as different levels of compliance with different recommendations were evident, this does not allow for standardised ‘usual care’ across the study. This does mean that usual practice can be compared over time within each trust individually, in order to assess how much ‘usual care’ has changed throughout the course of their participation in the STEPWISE study.

The Royal College of Psychiatrists' National Audit of Schizophrenia includes standards on the monitoring of physical health in patients with schizophrenia. The audit report in 2014 noted that the provision of interventions is poor when there is evidence of physical health risks.^14^ It highlighted barriers to intervention, such as availability of staff time, facilities and equipment, the need for formal systems to conduct annual reviews, and the need for more formal arrangements regarding responsibility for physical health between primary and secondary care.^14^ Standard 5 in the audit specifically looks at interventions offered for particular physical health risks. The overall results show that intervention for BMI>25 kg/m^2^ was evident in 71% of patients, while interventions for smoking were reported in 59% of patients.^14^

For the ten trusts surveyed, the audit reported a range of 47–83% of patients receiving intervention for elevated BMI, and 33–67% receiving intervention for smoking. Overall, the audit also showed wide variation in the monitoring of physical health risk factors. For example, the range across all participating trusts for monitoring of BMI was 5–92% of patients and 16–99% for the monitoring of glucose control. This is supportive of the information yielded from the study survey and highlights variability in services offered across trusts.^14^

### Implications for stakeholders

This survey indicates that the ten sites surveyed are not fully compliant with NICE physical health recommendations on the management of patients with schizophrenia. However, as the guidelines were published in March 2014, this is not surprising, because services require time to commission and set up. In some respects, this alleviates the concerns that the ‘usual care’ arm of the STEPWISE trial may converge with the intervention arm, as there are no reported standardised programmes offered across any of the trusts surveyed. However, there is such variability observed that it becomes clear that ‘usual care’ is not the same for all participants in the trial or in the wider population group.

We have not tried to assess the success or potential effect of any one aspect of the physical health programme in comparison with others. Although it may be argued that smoking cessation could have a greater effect on physical health than healthy eating or physical activity programmes, despite the lower compliance with NICE guidance, any discussion is likely to be subjective. Furthermore, NICE does not prioritise any one element, coming from an understanding that all aspects are important and contribute to improved physical health.

As a future direction, it may be useful to try to identify patients at higher risk of cardiovascular events, using recording of cardiovascular disease risk factors in combination with risk engines to calculate risk accurately. This identification process could then drive intervention. This may be of interest to trusts as a method of offering a physical health intervention to those patients who are likely to receive the most benefit clinically. A suitable risk engine to calculate this would be the PRIMROSE cardiovascular disease algorithm,^15^ as this has been developed specifically for people with severe mental illness. The PRIMROSE model includes additional variables for psychiatric diagnosis, psychotropic medication, harmful use of alcohol, antidepressants and social deprivation score, unlike similar prediction models used in the general population. This is perhaps why PRIMROSE performed better than some other available published risk models, which may overpredict the risk of cardiovascular disease in this population.^15^

### Further research

The survey will be repeated with the same ten NHS trust representatives at 12 and 24 months after the first iteration. This will allow the STEPWISE study team to consider how trusts are implementing the NICE guidelines and, consequently, whether convergence has occurred between the two arms of the STEPWISE trial at an individual trust level. As STEPWISE progresses, the participating NHS organisations may become more aware of the need to undertake physical health interventions and so ‘usual care’ may improve, potentially diminishing the effect of the STEPWISE intervention.
